# Resilience and Adaptation Among Ukrainians Across Two Cross‐Sectional Measurements Amid Prolonged Conflict

**DOI:** 10.1002/jcop.70122

**Published:** 2026-06-26

**Authors:** Shaul Kimhi, Mykola Nazarov, Hadas Marciano, Yohanan Eshel, Bruria Adini

**Affiliations:** ^1^ ResWell Research Collaboration, Gray Faculty of Medical and Health Sciences Tel Aviv University Tel Aviv Israel; ^2^ Stress and Resilience Research Center Tel‐Hai University of Kiryat Shmona in the Galilee Kiryat Shmona Israel; ^3^ Department of Psychology, Political science and socio‐cultural technologies Sumy State University Sumy Ukraine; ^4^ The Institute of Information Processing and Decision Making University of Haifa Haifa Israel; ^5^ Department of Emergency and Disaster Management, School of Public Health, Gray Faculty of Medical and Health Sciences Tel Aviv University Tel Aviv Israel

**Keywords:** coping mechanisms, prolonged war, PTSD symptoms, resilience, Ukraine

## Abstract

The Russian invasion of Ukraine transitioned from an “existential war” into a “war of attrition.” The study assessed the impact of prolonged conflict on resilience and coping mechanisms through two cross‐sectional assessments conducted with independent samples (Sample 1 – July 2022 [*N* = 1001]; Sample 2 – November 2023 [*N* = 2247]). The respondents completed a quantitative questionnaire during the war, which included individual, community, and societal resilience, hope, sense of danger, perceived threats, and posttraumatic stress disorder (PTSD). A significant decrease in all variables was found between Sample 1 and Sample 2 measurements. Path analysis indicated that government support is the variable most strongly related to societal resilience. Community resilience is the variable most strongly related to individual resilience. Perceived threats are the variable most strongly associated with PTSD symptoms. The above‐mentioned variables explain 68% (Sample 1) and 60% (Sample 2) of societal resilience, 19% (Sample 1 and Sample 2) of individual resilience, and 58% (Sample 1) and 12% (Sample 2) of PTSD symptoms. Findings suggest the “routinization” of an ongoing emergency, allowing the population to coexist with the adversity and accept war as the “new normalcy.” Nonetheless, the future remains uncertain, leading to a decline in hope. Strategies that bolster individual and societal resilience during adversities should include social support mechanisms that enhance the population's hope and morale and build the government's trust and support.

## 1 Introduction

War has inherent logic and development, progressing through distinct stages and transforming the battlefield landscape and the public's perception of the conflict (Gartner and Gelpi 2016; Chylińska 2020). The full‐scale invasion by the Russian Federation into Ukraine in 2022 was defined as an “existential war” or “a war for independence,” perceived as a struggle against a significant historical threat epitomized by the Russian Federation (Aliyev 2022; Kimhi et al. 2023). In July 2022, large‐scale missile attacks on civilian areas, such as the Serhiiyka strike in Odesa region and the Chasiv Yar apartment attack, caused mass casualties and destruction of homes (OCHA, 2022). These events intensified significant distress (symptoms of anxiety and depression) and a heightened sense of danger, but also triggered strong community‐level mobilization, humanitarian assistance, and solidarity networks. This period was marked by high levels of individual resilience (IR), societal (SR), and community resilience (CR), morale, and individual and collective hope (Kimhi et al. 2023; Goodwin et al. 2023; Reznikova and Korniievskyi 2024). Resilience signifies the capacity to function (during) and recover (even “bounce forward”) successfully from adversities, on the individual (IR), community (CR), and societal SR) level (Kimhi et al. 2023). The contradictory mosaic of a high and continuous risk resulting from the protracted war, along with societal cohesion, supported rapid self‐organization, strengthened civilian‐military cooperation, and enhanced community resilience (Lebid et al. 2022). Community resilience was particularly crucial at the onset of the invasion, suggesting a link between community strength and the ability to withstand military aggression. Community resilience is understood as the collective capacity of communities to withstand, adapt to, and recover from adversity through resources such as social capital, trust, leadership, and communication networks (Patel et al. 2017). In contexts of protracted conflict, strong social cohesion and volunteerism function as mechanisms of coping by mobilizing and conserving shared resources, thereby enabling rapid self‐organization and sustained adaptation (Hobfoll et al. 2015). In Ukraine, studies document that these communal coping mechanisms have been central to maintaining resilience under prolonged threat (Reznikova and Korniievskyi 2024; Palgi et al. 2023), of community resilience and directly affect Ukraine's territorial communities' capacity and effectiveness to cope with the attempts at occupation (Goodwin et al. 2023; Kupenko et al. 2023).

Since mid‐2023, the conflict has shifted into what is termed a “war of attrition” (Reznikova and Korniievskyi 2024; Turchin n.d.). By November 2023, the resilience of individuals and communities was tested by intensified Russian strikes on Ukraine's energy, water, and heating infrastructure, particularly critical before winter. Nearly 3,800 schools and over 1,300 health facilities were damaged or destroyed, disrupting education and medical care for millions (OCHA, 2023). Communities adapted by relying on online/hybrid education, emergency healthcare, home repair, and winterization measures such as heating support (Albakjaji 2023; Lebid et al. 2023). International and local aid networks delivered food, cash, and winter supplies to millions, highlighting the interplay between external assistance and community resilience (Kimhi et al. 2023; Lebid et al. 2022). Resilience at this stage has been challenged by prolonged uncertainty, resource depletion, and the normalization of war conditions. Communities and individuals increasingly relied on adaptive coping, digital networks, and volunteer coordination to sustain everyday life despite stalemate conditions (Reznikova and Korniievskyi 2024; Eshel et al. 2023).

As 2023 progressed, Russia intensified its efforts to undermine societal resilience by targeting Ukraine's internal cohesion (Crock et al. 2015; Figueiredo and Petraviciute 2025; Figueiredo et al. 2024; Figueiredo and Ndiaye 2025; Wang et al. 2024; An et al. 2025). Lacking formal political allies inside Ukraine, Russia sought instead to undermine resilience by exploiting social stressors, such as the burdens of mobilization, declining living standards, fear, and diminishing hope (Aliyev 2022). Russia's objective was to foster political and social destabilization within Ukraine (Stoicescu et al. 2023), and thus damage the societal resilience of Ukraine's population. The evolution of military operations in Ukraine from Russia's initial invasion in February 2022 to the latter half of 2023 reflects a significant shift in the conflict's dynamics. This observation leads us to hypothesize a transition from a “war for independence” to what could be termed as “routinization of the war” (Eshel et al. 2023; Otrishchenko 2023). A distinct division within society marks this new phase. On one side are those actively engaged in the war effort—including military personnel, their families, volunteers, and officials. On the other hand, some individuals have become passive or indifferent, regarding the ongoing war as the new normal.

Drawing on earlier research findings (Kimhi et al. 2023; Eshel et al. 2023), we anticipate that by the second half of 2023, measures of the three types of resilience (individual, community, and societal) will begin to decline, as prolonged exposure to conflict, economic hardship, and mobilization fatigue gradually erode morale, hope, and collective coping capacities. Correspondingly, we expect a decrease in positive coping mechanisms (such as morale and hope). We can also expect stabilization or reduction in negative coping mechanisms (like the sense of danger, distress, post‐traumatic stress disorders [PTSD], and perceived threats) attributed to the psychological acclimatization of individuals to the ongoing conflict as a normalized aspect of life. PTSD was included as a mechanism of poor coping, as it reflects trauma‐related distress and broader disruptions in functioning that challenge individual resilience. The November 2023 humanitarian situation in Ukraine, marked by winter hardships, infrastructure damage, and large‐scale displacement, illustrates both the strain on resilience capacities and the adaptive capacities—material, psychological, and communal—that allow Ukrainian society to endure the protracted war. Studies highlight elevated PTSD symptoms among internally displaced persons (IDPs), refugees, and civilians who remained in heavily affected areas of Ukraine, with IDPs showing particularly severe outcomes (Lushchak et al. 2024; Rizzi et al. 2023). PTSD prevalence is especially high in regions of geopolitical significance contested by Russia, such as Donetsk, Luhansk, and Crimea, where repeated occupation attempts and chronic insecurity intensify trauma exposure and displacement (Brackstone et al. 2024). In these contexts, coping functions as a mechanism rather than a fixed strategy, mediating between continuous threat and psychological outcomes, with avoidant or emotion‐focused coping linked to poorer adaptation and community‐level coping processes—such as mutual aid and collective action ‐ supporting resilience (Rizzi et al. 2023).

The theoretical model evaluated in this study was designed to elucidate the dynamic interplay between various coping mechanisms, resilience, and PTSD symptoms among the Ukrainian population during the ongoing conflict. The model integrates psychological and environmental variables to forecast three primary outcomes: individual resilience, societal resilience, and PTSD symptoms. The model categorizes variables into positive (morale, hope, and community resilience) and negative (perceived threats, distress symptoms, and a sense of danger) coping mechanisms and includes governmental support as an environmental variable. The primary outcomes assessed are two forms of distinct types of resilience (IR and SR) and PTSD symptoms. The model posits that optimistic and proactive attitudes foster better psychological adaptation, and continuous exposure to perceived threats and distress increases vulnerability to PTSD and erodes resilience. At the same time, robust support of the government strengthens resilience by providing stability, security, and resources necessary for coping with the war's impact. The model also accounts for interactions among the varied variables. This theoretical framework is grounded in the resilience literature, which emphasizes the role of internal psychological states and external support systems in coping with adversity (Carver and Connor‐Smith 2010). The model aims to provide insights into the mechanisms underpinning resilience and psychological distress during prolonged conflicts by analyzing how these variables interact over time. The model is in line with the recommendations of Kalisch et al. (Kalisch et al. 2021) that posit that resilience needs to be periodically monitored over time, along with the exposure to stressors that occur during adversities. They further stress that rather than concentrating on the underlying pathophysiology of stress‐related conditions, resilience research should emphasize the protective mechanisms that help individuals resist developing mental health disorders despite severe adversities (Kalisch et al. 2017). Thus, the two main objectives of the current study were: (a) to assess and compare the changes over time in individual, community, and societal resilience, as well as positive and negative coping mechanisms and government support within the Ukrainian population, based on data from the first (July 2022) and second (November 2023) measurements. This comparison aims to elucidate the impact of a prolonged conflict on these variables. (b) to identify coping mechanisms that are most highly associated with societal resilience, individual resilience, and PTSD symptoms. We built upon variables from an earlier study conducted during the ongoing war in Ukraine (Kimhi et al. 2023; Eshel et al. 2023). We retained the same core variables so that results would be comparable across time. The first measurement occurred a few months after the invasion, while the current study took place a year and a half later, enabling us to examine longitudinal changes in coping mechanisms under extended war conditions. This previous research identified three variables ‐ societal and individual resilience and PTSD symptoms – as significantly related to individual coping mechanisms in war conditions. Based on these findings, we hypothesized that similar resilience and coping mechanisms across both measurement periods in Ukraine would influence the same variables.

## 2 Method, Sample, and Sampling

The study is based on cross‐sectional assessments of two independent samples of the Ukrainian population residing in varied regions of Ukraine, excluding Crimea and the areas at the time of data collection under Russian occupation (Donetsk and Lugansk regions). Data collection for both measurements (Sample 1 [*N* = 1001] and Sample [*N* = 2247]) was conducted by the same Ukrainian internet panel company, which sampled diverse sectors of the population. The characteristics of the study population in the two measurements are presented in Table 1. Data was collected between July 18 and 28, 2022 (Sample 1) and November 1 and 13, 2023 (Sample 2). All respondents provided informed consent before completing the questionnaire. All research methods were carried out in accordance with institutional and national guidelines and regulations. The study was ethically approved by the Ethics Committee of Tel Aviv University (#0005146, issued on July 12, 2022).

**Table 1:** Comparison of the demographic characteristics of the Ukrainian samples across two independent measurements.

		Sample 1 (*N* = 1001) July 2022			Sample 2 (*N* = 2,247) December 2023			*F*	Effect size Eta^2^
Variable (scale)	Categories (% according to State Statistics Service of Ukraine)	*N*	%	*M* (S.D.)	*N*	%	*M* (S.D.)		
Age groups (1–5)	1. 18–25 (15%)	139	13.9	37.26 (9.56)	277	12.3	42.17 (13.3)		
	2. 26–35 (17%)	279	27.9		483	21.5			
	3. 36–45 (18%)	351	35.1		585	26.0		110.65***	0.033
	4. 46–55 (24%)	232	23.2		460	20.5			
	5. 56 and older (26%)	—	—	—	442	19.7			
Education (1–5)	1. Elementary (4%)	3	3	4.03 (0.95)	13	0.6	3.79 (1.04)		
	2. High school (7%)	52	5.2		213	8.6		38.16***	0.012
	3. More than high (25%)	264	26.4		786	35.0			
	4. Bachelor's degree (33%)	278	27.8		458	20.4			
	5. Master's & above (31%)	404	40.4		777	34.6			
Gender	Male (45%)	489	48.9	—	1370	52.1			
	Female (55%)	512	51.2	—	1077	47.9			
Family income (1–5)	1. Below (43%)	494	49.4	2.49	1574	70.0	2.35		
	3. Average (42%)	335	33.5	(1.04)	589	26.2	(1.01)	13.82***	0.004
	5. Above (15%)	173	17.3		84	3.7			
Political attitudes (1–5)	1. Left (41%)	32	3.2		246	10.9			
	3. Center (30%)	237	23.7		589	26.2		0.007	0.000
	5. Right (24%)	107	10.7		84	3.7			
	0. Difficult to answer (5%)	625	62.5		1401	62.3			
Religiosity (1–4)	Secular (15%)	221	22.1	2.05 (0.73)	348	15.5	2.18 (0.73)		
	Traditional (10%)	531	53.1		1226	54.6		22.25***	−0.007
	Religious (65%)	226	22.6		589	26.2			
	Orthodox (10%)	232	23.2		84	3.7			
	1. up to 5,000 (5%)	42	4.2		621	27.6			
Community	4. up to 50,000 (15%)	40	4.0	7.72	551	24.5	5.77	480.28***	0.129
Size (1–9)	9. up to 1,000,000 (80%)	919	91.8	(1.44)	1075	47.8	(2.64)		

***p < 0.001, asterisks mean that the findings are significant.

## 3 Tools

The study was designed in English and translated into both Ukrainian and Russian, so that each respondent could choose the language most convenient to him/her. The translations from English to each of the other languages were done back and forth to ensure accuracy. The study was based on a structured quantitative questionnaire that included the following sections:

(1) Distress symptoms (BSI, (Kimhi and Eshel 2019). Four items relating to anxiety (e.g.,: “I feel such restlessness that it is impossible to sit in one place”) and four items relating to depressive symptoms (such as “I feel a lack of interest in my world”) were included, ranked on a 5‐point Likert scale, ranging from 1 = not at all to 5 = to a considerable extent. The internal reliability, measured by Cronbach's alpha of the scale, was excellent (Sample 1 *α* = 0.89, Sample 2 *α* = 0.89).

(2) Societal resilience (SR) (Kimhi and Eshel 2019). Ten items (such as “Ukraine is my home, and I do not intend to leave it”) ranged from 1 = strongly disagree to 6 = strongly agree. The scale has been used in various countries during the COVID‐19 pandemic, including Brazil and the Philippines, and has been shown to be valid and reliable. The internal reliability, measured by Cronbach's alpha of the scale, was excellent (Sample 1 *α* = 0.91, Sample 2 *α *= 0.89).

(4) Community resilience (CR) (Leykin et al. 2013). Seven items (such as: “I can trust people in my community to come to my aid in case of crisis”) ranged from 1 = do not agree at all to 5 = agree to a considerable extent. The scale has been used in numerous previous studies conducted in varied countries (McNeill et al. 2022). The scale's internal reliability, as measured by Cronbach's alpha, was excellent (Sample 1: *α* = 0.90; Sample 2: *α* = 0.90).

(5) Individual resilience (IR) (Connor and Davidson 2003). The two items (such as: “I can adapt when changes occur”) suggested by the tool's authors were used, ranging from 0 = do not agree at all to 4 = agree to a considerable extent (for the analysis of the data, we have re‐coded the scale to 1–5). This Connor‐Davidson brief (two‐item) scale is widely used in numerous countries to measure IR and its internal consistency was found to be moderate and appropriate at the level of 0.66 (Kuiper et al. 2019). The internal reliability, measured by Cronbach's alpha, was acceptable for the two‐item scale (Sample 1 *α* = 0.67, Sample 2 *α* = 0.66).

(6) Hope (Jarymowicz and Bar‐Tal 2006; Marciano et al. 2022). Three items were adapted in their context to a security threat (e.g.,: “I have hope that I will emerge strengthened from the Ukraine war”), ranging from 1 = very little hope to 5 = very much hope. The scale's internal reliability, measured by Cronbach's alpha, was good (*α* = 0.80).

(7) Morale. We have used one item: “What is your morale (personal mood) these days?” The question was answered on a 5‐point Likert scale, ranging from 1 = very bad to 5 = very good. This item was previously used in other studies and found to be valid (Eshel et al. 2021).

(8) Sense of danger (Solomon and Prager 1992; Kimhi et al. 2020). Four items were included (e.g., “To what extent do you feel that your life is in danger due to the war in Ukraine?”), ranging from 1 = not at all to 5 = to a very large extent. This scale was previously used in other studies and found to be valid and reliable (Solomon and Prager 1992; Kimhi et al. 2020) The scale's internal reliability, measured by Cronbach's alpha, was acceptable (Sample 1 *α* = 0.78, Sample 2 *α* = 0.77).

(9) Perceived threats (Kimhi and Eshel 2012). Five items relating to five types of adversities were included—economic, social, security, political, and health risks. The responses were given on a 5‐point Likert scale, ranging from 1 = not threatening at all to 5 = threatening to a considerable extent. The scale's internal reliability, as measured by Cronbach's alpha, was good (Sample 1: *α* = 0.84; Sample 2: *α* = 0.82).

(10) PTSD symptoms (Lang and Stein 2005). An abbreviated PTSD checklist for use as a screening instrument in primary care was utilized (e.g., “In the past month, how much have you been bothered by repeated, disturbing memories, thoughts, or images of a stressful experience from the past.” The scale consists of six items, ranging from 1 = not at all to 5 = extremely threatening. This screening measure was selected, rather than a more comprehensive measure (such as the PCL‐5), to minimize participant burden, maximize response rates, and enhance feasibility for large‐scale data collection. The scale's internal reliability, measured by Cronbach's alpha, was excellent in the first and acceptable in the second (Sample 1 *α* = 0.91, Sample 2 *α* = 0.75). The decrease in alpha from Sample 1 to Sample 2 may be attributed to the larger and more heterogeneous sample at Sample 2 (*N* = 2247 vs. *N* = 1001 at Sample 1), which likely reduced inter‐item correlations. Importantly, the T2 value still reflects acceptable reliability.

(11) Demographic characteristics included 13 items: age, gender, socioeconomic status, education, political attitudes, level of religiosity, country of birth, place of residence, size of community, marital status, number of children, support of the government, and level of exposure to the war. As can be seen in Table 1, significant differences were found in several demographic characteristics between the two samples (Sample 1 and Sample 2): (a) age in Sample 2 was significantly higher than in Sample 1 (in Sample 1, there were no participants older than 56). (b) Education in Sample 1 was significantly higher than that in Sample 2. (c) Sample 1 participants reported a significantly higher average family income than Sample 2 participants (this was an effect with a very small effect size – ETA^2^ = 0.004). (e) Religiosity in Sample 2 was significantly higher compared to Sample 1. (f) A higher percentage of participants reported living in the same place before the war in Sample 2 compared to Sample 1. (g) Sample 1 participants lived in larger communities compared with Sample 2 participants. However, most differences showed small effect sizes.

### 3.1 Statistical Analysis

The statistical analyses were completed by SPSS, version 29 (IBM Corp. (2022). IBM SPSS Statistics for Windows, version 29.0 [Computer software]. Armonk, NY: IBM Corp). First, using analysis of variance (ANOVA), we examined the differences between the samples that participated in the two measurements in Ukraine regarding the ten examined variables: individual, community, and societal resilience, morale, hope, sense of danger, distress symptoms, PTSD symptoms, perceived threats, and level of government support. ANOVA was used instead of a *t*‐test because it allowed simultaneous comparison of repeated measures and group characteristics across the two independent groups. Second, we examined the correlations between the study variables in the two measurements. Third, using path analysis (Arbuckle 2011; Falakshahi et al. 2022), we identified variables associated with individual resilience, societal resilience, and PTSD symptoms in Samples 1 and 2 using SPSS‐AMOS (version 30).

## 4 Results

Results indicated significant differences across all variables (except distress symptoms) between the two measurements. The following scales, which represent both positive and negative coping, decreased significantly between Sample 1 and Sample 2: IR, CR, SR, level of hope, sense of danger (the largest decline), PTSD symptoms, and level of government support. The only scale that increased significantly was morale. Overall, most positive coping mechanisms (including the three types of resilience and hope) and all negative coping mechanisms, as well as government support, decreased (Table 2).

**Table 2:** Comparison between the two samples of Ukrainians: resilience, coping mechanisms, and government support.

Area	Variable	Scale range	Ukraine measurement 1 *N* = 1001			Ukraine measurement 2 *N* = 2247			*F*	Effect size Eta^2^
			*M*	S.D	*α* ^2^	*M*	S.D	α^2^		
Resilience	IR.	1–5	3.648	0.755	0.67	3.586	0.810	0.66	4.25*	0.001
	CR.	1–5	3.405	0.736	0.67	3.096	0.824	0.66	103.67***	0.031
	SR.	1–6	4.349	0.972	0.89	3.898	1.033	0.90	136.83***	0.040
Positive coping	Morale (1 item)	1–5	2.901	0.786	—	3.000	0.829	—	9.70**	0.003
	Hope	1–5	3.955	0.924	0.88	3.739	1.016	0.86	33.00***	0.010
Negative coping mechanisms	Danger	1–5	3.699	0.769	0.78	2.275	0.762	0.77	2398.12***	0.425
	Distress symptoms	1–5	2.946	0.879	0.89	2.890	0.867	0.89	2.83	0.001
	PTSD	1–5	2.892	0.962	0.91	2.252	0.849	0.75	361.44***	0.100
	Perceived threats	1–5	3.216	0.886	0.84	3.089	0.900	0.82	13.81***	0.004
Government support (1 item)		1–5	3.568	1.059	—	2.974	1.150	—	193.88***	0.056

*p < 0.05, **p < 0.01, ***p < 0.001, asterisks mean that the findings are significant.

Pearson's correlation matrix was used to examine the similarity of the associations among the study variables across the two measurements (see Table 3). Overall, the results indicated that the correlations between the same variables at Sample 1 and Sample 2 were generally consistent in both direction and magnitude. Most associations fell within the small to moderate range (*r* = 0.10–.40), whereas larger effects emerged for conceptually related constructs, such as PTSD symptoms and distress (Sample 1 *r* = 0.74; Sample 2 *r* = 0.29), and societal resilience with government support (Sample 1 *r* = 0.69; Sample 2 *r* = 0.65). Notably, only one pair of variables, distress symptoms and sense of danger, shifted from a positive correlation at Sample 1 (*r* = 0.44) to a negative correlation at Sample 2 (*r* = −0.28), indicating a meaningful change in the nature of the relationship. Three additional variable pairs showed varying degrees of correlation across measurements, though all remained within the small to moderate range.

**Table 3:** Pearson correlations among the research variables across the two measurements.

Variable	Time	2	3	4	5	6	7	8	9	10. Government support
Predictors										
1. Societal resilience	S1	0.200^1^	0.061	0.621^1^	−0.105	**−0.004**	−0.176^1^	0.178^1^	0.593^1^	0.692^1^
	S2	0.196^1^	0.018	0.557^1^	−0.092^1^	**0.050** ^**2**^	−0.204^1^	0.258^1^	0.405^1^	0.650^1^
2. Individual resilience	S1		−0.263^1^	0.286^1^	−0.284^1^	−0.067^1^	−0.121^1^	0.316^1^	0.305^1^	0.123^1^
	S2		−0.029	0.324^1^	−0.210^1^	0.028	−0.086^1^	0.295^1^	0.312^1^	0.086^1^
3. Posttraumatic symptoms	S1			−0.074^2^	0.738^1^	0.395^1^	0.490^1^	−0.510^1^	**−0.089** ^**2**^	0.051
	S2			0.028	0.286^1^	−0.223^1^	0.236^1^	−0.065^2^	**−0.017**	−0.031
4. Community resilience	S1				−0.130^1^	0.022	−0.112^1^	0.215^1^	0.397^1^	0.422^1^
	S2				−0.124^1^	0.035	−0.193^1^	0.318^1^	0.367^1^	0.339^1^
5. Distress symptoms	S1					**0.443** ^**1**^	0.476^1^	−0.578^1^	−0.185^1^	**−0.061**
	S2					**−0.276**	0.386^1^	−0.461^1^	0.187^1^	**−0.122** ^**1**^
6. Sense of danger	S1						0.515^1^	−0.339^1^	−0.012	0.043
	S2						0.425^1^	0.174^1^	0.013	0.027
7. Perceived threats	S1							−0.378^1^	−0.149^1^	−0.098^2^
	S2							−0.235^1^	−0.122^1^	−0.173^1^
8. Morale	S1								0.244^1^	0.095^2^
	S2								0.301^1^	0.195^1^
9. Hope	S1									0.381^1^
	S2									0.302^1^

^1^ *p* < 0.001, ^2^ *p* < 0.05, Bolded pairs of correlations indicate a discrepancy between the variables' correlations in the two measurements.

**Figure 1:**
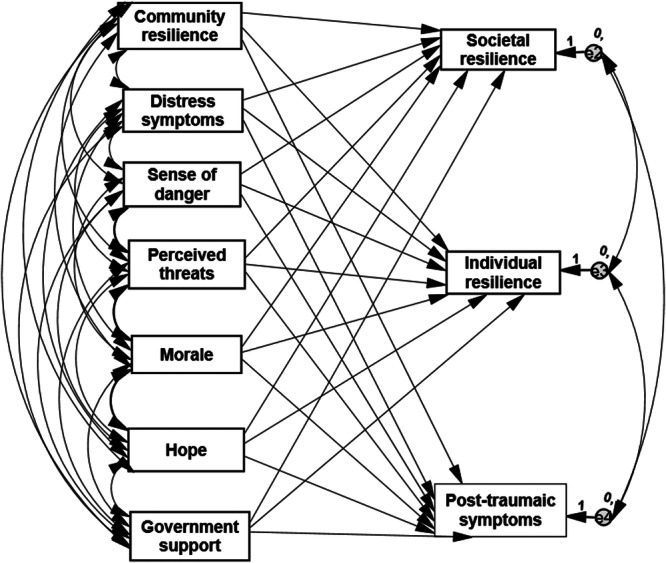
Theoretical model: Path Analysis of coping mechanisms and level of government support as predictors of societal and individual resilience and post‐traumatic symptoms, among two independent samples of the Ukrainian population (Sample 1 *N* = 1001 and Sample 2 *N* = 2247).

Path analysis (a statistical technique to understand the relationships between variables) was used to examine whether similar coping mechanisms and levels of government support explain SR, IR, and PTSD symptoms in both measurements. We used a saturated model that examined all paths and correlations, and thus, there is no need to determine the model fit (see Table 4 and Figure 1) (Arbuckle 2011). We selected a saturated model because our aim was to estimate all possible associations among the study variables across time, rather than to test the fit of a constrained theoretical model. Saturated models, which have zero degrees of freedom, fit the data completely and are meaningful in exploratory contexts where the focus is on parameter estimates rather than global fit indices (Kline 2023). Results show the following: (a) The variable most strongly associated with SR is the level of government support, followed by community resilience and hope in both measurements. (b) The variables most strongly positively associated with IR are levels of community resilience, hope, and morale, and the variable most strongly negatively associated is distress symptoms in both measurements. (c) The variable most strongly associated with PTSD symptoms is perceived threats in both measurements. Nonetheless, distress symptoms, a sense of danger, and community resilience were also found to contribute to PTSD symptoms in the second measurement, while hope contributed to the first measurement. Morale contributed to both measurements, though the association was negative in the first and positive in the second. (d) The associated variables explain 68% (at Sample 1) and 60% (at Sample 2) of societal resilience, 19% (at Sample 1 and Sample 2) of individual resilience, and 58% (at Sample 1) and 12% (at Sample 2) of PTSD symptoms. Among the three explained variables, PTSD symptoms are the only index that exhibits a substantial difference between the two measurement points.

**Table 4:** Standardized path analysis predicting resilience and PTSD symptoms.

Time of measurement	Predictor	Societal resilience estimate	Individual resilience estimate	PTSD estimate
S1	Community resilience	0.316***	0.177***	0.031
S2		0.300***	0.231***	0.065**
S1	Distress symptoms	0.057	−0.173***	0.003
S2		0.083***	−0.110***	0.252***
S1	Sense of danger	−0.042	−0.015	0.003
S2		0.020	−0.020	−0.120***
S1	Perceived threats	−0.074***	0.049***	0.167***
S2		0.035	−0.053	0.116***
S1	Morale	0.006	0.170***	−0.117***
S2		0.019	0.141***	0.081***
S1	Hope	0.300***	0.187***	0.075***
S2		0.245***	0.195***	0.002
S1	Government support	0.441***	−0.047	−0.029
S2		0.471***	−0.085***	−0.015
S1	% variability	0.686	0.195	0.582
S2	% variability	0.604	0.189	0.123

*Note:* Bolded are pairs of correlations that indicate a discrepancy between the variables' correlations in the two measurements; asterisks mean that the findings are significant.

## 5 Discussion

These two cross‐sectional assessments of two independent samples examined the temporal changes in resilience (individual, community, and societal), well‐being, and coping mechanisms among the Ukrainian population during the prolonged war with Russia. Furthermore, it explored the factors that influence individual and societal resilience alongside PTSD symptoms. The findings reveal a significant decrease in all of the positive indices (the three types of resilience and hope, excluding morale), as well as in all negative coping mechanisms (sense of danger, perceived threats, and PTSD symptoms), during the second measurement compared to the levels that were identified in the earlier stage of the war. Nonetheless, due to distinct differences between the two samples that were examined in 2022 and 2023, the assessment of longitudinal change should be interpreted with caution, as the design does not permit direct within‐person comparisons.

The notable increase in morale, contrasted with the decline in all the other variables, suggests a complex and uneven pattern of adaptation over time, indicating that certain adaptive capacities were strengthened while others weakened. It seems that a ‘routinization’ of the ongoing emergency is emerging among the population (Otrishchenko 2023), allowing them to continue their lives even though the war has not yet been resolved and attacks against civilian communities still pose a realistic risk. The varied everyday occurrences experienced over the war's 2 years link the past, the present, and the future, leading to the expectation that the future will unfold in familiar patterns that seem less daunting as they are known (Bakonyi 2022). Though the war is in its midst, the Ukrainian population may have learned to coexist with this adversity and created internal dialogues to regain control over their situation, including routinizing the war's consequences and accepting them as a “new normalcy” (Makarychev 2023). The above enables them to practice acceptance, make downward social comparisons, and engage in daily activities, leading to a decreased sense of danger, perceived threats, and PTSD symptoms, with a concurrent rise in the level of morale. The perceived “success” in overcoming the many complexities that challenge the population most probably contributes to their current belief that they can overcome these challenges at this time, and consequently, the morale increases (Rizzi et al. 2023; Brackstone et al. 2024; Kurnyshova and Makarychev 2023). Nonetheless, looking ahead, the future is still perceived as bleak and uncertain, which explains the significant decline in hope (Driscoll and Savelyeva 2023). Similarly to the Ukrainian population, adaptation of a “new normalcy,” due to protracted conflicts, was also identified among Afghan and Iraqi populations (Policinski and Kuzmanovic 2019), Syrian refugees (Dami 2018), and Nigeria (El Jurdi et al. 2025).

Previous research findings have suggested an inverse association between negative coping mechanisms (such as a sense of danger or perceived threats) and resilience (Salceanu 2022; Kimhi et al. 2023). Conversely, the current study shows that such negative coping mechanisms that decrease over time may align with similar trends in decreased individual, community, and societal resilience. A potential explanation for the decline in negative coping mechanisms was previously suggested, positing that the population has become familiar with the threat and is less frightened than at the onset of the crisis (Pavlenko 2023). Accordingly, the perception of reality changed after having survived the war for an extended period. Nonetheless, it does not necessarily enhance capacity to function during wartime or a more effective capability to “bounce forward” or be empowered by the crisis (Driscoll and Savelyeva 2023).

Identifying variables that are associated and explain individual and societal resilience elucidates factors influencing adaptive responses during varied crises. Supporting the government emerged as a pivotal variable associated with societal resilience, underscoring the role of governance systems in enhancing the population's capacity to cope with adversity (O'Grady and Shaw 2023). Similar findings also highlight the vital role of government support in improving the quality of life of affected populations, as demonstrated by the displaced Maguindanaon women (Haron and Alongan 2023).

In contrast, in the current study, government support was found to have only a negligible impact (if any) on individual resilience. Other variables, such as hope, morale, and community resilience, contributed substantially to the individual's capacity to cope with the ongoing war crisis. Hope refers to the belief in positive outcomes (in the coming periods) despite the challenges created by the ongoing war, while morale encompasses the current emotional and psychological well‐being (Kimhi et al. 2024). Individuals who maintain high levels of hope during crises are more likely to exhibit resilience by actively seeking solutions, maintaining optimism, and adapting to adverse circumstances (Leslie‐Miller et al. 2023). These psychological factors serve as internal resilience mechanisms that empower individuals to navigate and cope with the various stressors created by the war. Heightened levels of hope and morale may buffer against stress (Kimhi et al. 2023).

As previously reported in various studies, hope and community resilience were also identified in our study as vital components influencing societal and individual resilience during adversity (Kimhi et al. 2023). For example, Ukrainian respondents had substantially higher levels of hope and resilience than those from other countries, who were not as exposed to similar existential threats, suggesting a form of adaptation to the challenging situation (Kimhi et al. 2023; Kimhi et al. 2024; Goodwin et al. 2023; Esbit et al. 2025).

The current study found that community resilience is the most highly associated with individual resilience. Community resilience reflects the collective capacity of social networks and community structures to provide support, resources, and solidarity during conflicts. Strong community ties and mutual support mechanisms enhance individuals' ability to cope with stress and adversity. Individuals embedded in supportive communities frequently benefit from shared coping strategies, emotional support, social capital, and practical assistance, which are pivotal for maintaining resilience in challenging contexts (Kupenko et al. 2023; Salceanu 2022).

Perceived threats were the only consistent factors that were found to predict PTSD symptoms in both measurements of the current study. Morale was found to be an additional contributor to PTSD symptoms, though the association was negative in the first assessment and found to be positive in the second measurement. This shift may result from adaptation over time during the protracted war. In the initial phase, individuals with higher morale may have been better equipped to cope with the stresses of war, thus suffering from fewer PTSD symptoms (Whitesell and Owens 2012). However, as the war progressed, the prolonged exposure to traumatic events may have overwhelmed the initial protective effects of high morale (Al Jowf et al. 2022). The emotional and psychological exhaustion that frequently develops in prolonged traumatic situations may lead to a decrease in the impact of morale on positive mental health (Williamson et al. 2020). Similar declines in mental health were identified among individuals from many other conflict‐ridden societies, presenting a prevalence of distress and PTSD that was two to three times higher compared to populations who were not exposed to such circumstances (Eshel et al. 2023; Figueiredo and Petraviciute 2025). In the second measurement, distress symptoms, along with a sense of danger, were also found to contribute to the formulation of PTSD symptoms. Nonetheless, the overall explanation of PTSD symptoms in the second versus the first measurement was much lower (overall explanation of 12% vs*.* 58% of the PTSD symptoms, respectively). A plausible explanation is that the perceived risk, rather than the risk itself, impacts the individual's well‐being and ability to maintain an effective equilibrium during a crisis (Karagöz et al. 2023). Neither the actual (objective) severity of the situation nor its potential impact is likely to lead to the development of negative psychological coping mechanisms. Still, the severity and effects, as perceived by individuals, may lead to such repercussions.

### 5.1 Limitations

Despite its contributions, this study is not without limitations. The reliance on self‐report measures introduces potential biases, and the sample's specific geographic and temporal context may limit its generalizability. Future research should incorporate robust longitudinal designs to capture dynamic changes in resilience over time and include diverse populations to enhance the applicability of resilience models across various crisis scenarios.

The study design, consisting of two cross‐sectional assessments of independent samples, is a substantial limitation. Comparing the demographic data between the two samples revealed population differences, most notably in age. These differences require us to be cautious about the representativeness of the two samples despite their large size and their impact on the variability of resilience and coping mechanisms. The differences in demographic characteristics between the two samples could be influenced by several factors, including the second sample being more than twice the size of the first. A larger sample size may introduce greater variability and potentially capture a broader cross‐section of the population, including more diverse demographic groups. These distinct differences between the two samples limit the ability to compare 2022 and 2023 data.

Furthermore, the data collection for the two samples occurred at different times. Over this period, various demographic shifts may have occurred due to socioeconomic changes, migration patterns (including internal displacement and emigration), or other demographic trends influenced by ongoing conflict and economic conditions. Younger individuals, for instance, might have different migration patterns than older adults or be more likely to be involved in conflict, either directly or through displacement. These factors suggest that, even with a consistent sampling method, sample characteristics may vary significantly depending on external conditions and the dynamics of the studied population.

The resilience measures used in the study also have limitations, as they do not capture the complexity of this dynamic construct in relation to the stressor reactivity as a way to fully operationalize resilience (as delineated by Kalisch et al.) (Kalisch et al. 2021; Kalisch et al. 2017).

## 6 Conclusions

The study's findings offer several insights that should inform policymaking to enhance individual and societal resilience and mitigate psychological distress during prolonged crises. Strategies that focus on bolstering individual, community, and societal resilience during adversity, especially amid a prolonged human‐made conflict, should include social support mechanisms that enhance hope and morale across diverse population groups and build the government's trust and support. Furthermore, targeted interventions that address perceived threats and adapt mechanisms to evolving stressors are essential for effective crisis management and resilience.

This study contributes to community psychology by underscoring the multilevel nature of resilience in the context of protracted conflicts. The findings show that adaptive capacities are shaped not only by individual coping but also by community support and trust in governmental institutions, aligning with the ecological framework central to the field. The observed routinization of adversity highlights how communities adapt collectively to chronic stressors, even as hope for the future declines. These insights suggest that interventions in community psychology should focus on strengthening community solidarity, enhancing social support systems, and fostering institutional trust, while also promoting hope and morale as critical psychological resources.

In conclusion, this study contributes valuable insights into the complex interplay of resilience factors and coping mechanisms during crises. By elucidating the dynamics of resilience and psychological distress, the findings direct targeted efforts and policies to address perceived threats and foster adaptive responses to enhance societal resilience in the face of ongoing challenges.

## Author Contributions

**Shaul Kimhi** and **Bruria Adini:** conceptualization, methodology, writing – original draft preparation. **Shaul Kimhi** and **Mykola Nazarov:** software, writing – review and editing. **Shaul Kimhi** and **Bruria Adini:** validation. **Yohanan Eshel, Hadas Marciano:** formal analysis. **Shaul Kimhi:** investigation, resources, **Yohanan Eshel, Hadas Marciano,** and **Bruria Adini:** All authors have read and agreed to the published version of the manuscript.

## Funding

1

The authors have nothing to report.

## Ethics Statement

The study was ethically approved by the Ethics Committee of Tel Aviv University (#0005146‐1 from July 12, 2022). Data was collected anonymously.

## Conflicts of Interest

The authors declare no conflicts of interest.

## Data Accessibility Statement

Data supporting the findings of this study are available from the corresponding author upon reasonable request.
